# Long-term monitoring of COVID-19 prevalence in raw and treated wastewater in Salvador, the largest capital of the Brazilian Northeast

**DOI:** 10.1038/s41598-023-41060-1

**Published:** 2023-09-14

**Authors:** Carolina de Araújo Rolo, Bruna Aparecida Souza Machado, Matheus Carmo dos Santos, Rosângela Fernandes dos Santos, Maísa Santos Fonseca, Katharine Valéria Saraiva Hodel, Jéssica Rebouças Silva, Danielle Devequi Gomes Nunes, Edna dos Santos Almeida, Jailson Bittencourt de Andrade

**Affiliations:** 1 SENAI CIMATEC, SENAI Institute of Innovation (ISI) in Health Advanced Systems (CIMATEC ISI SAS), University Center SENAI/CIMATEC, Salvador, 41650-010 Brazil; 2 SENAI CIMATEC, Manufacturing and Technology Integrated Campus, University Center SENAI CIMATEC, Salvador, 41650-010 Brazil; 3 Centro Interdisciplinar de Energia e Ambiente – CIEnAm, Federal University of Bahia, Salvador, 40170-115 Brazil

**Keywords:** Biological techniques, Environmental sciences, Diseases, Chemistry

## Abstract

Wastewater-based epidemiology (WBE) becomes an interesting epidemiological approach to monitoring the prevalence of SARS-CoV-2 broadly and non-invasively. Herein, we employ for the first time WBE, associated or not with the PEG 8000 precipitation method, for the detection of SARS-CoV-2 in samples of raw or treated wastewater from 22 municipal wastewater treatment stations (WWTPs) located in Salvador, the fourth most populous city in Brazil. Our results demonstrate the success of the application of WBE for detecting SARS-CoV-2 in both types of evaluated samples, regardless of the usage of PEG 8000 concentration procedure. Further, an increase in SARS-CoV-2 positivity rate was observed in samples collected in months that presented the highest number of confirmed COVID-19 cases (May/2021, June/2021 and January/2022). While PEG 8000 concentration step was found to significantly increase the positivity rate in treated wastewater samples (*p* < 0.005), a strong positive correlation (r: 0.84; *p* < 0.002) between non-concentrated raw wastewater samples with the number of new cases of COVID-19 (April/2021–February/2022) was observed. In general, the present results reinforce the efficiency of WBE approach to monitoring the presence of SARS-CoV-2 in either low- or high-capacity WWTPs. The successful usage of WBE even in raw wastewater samples makes it an interesting low-cost tool for epidemiological surveillance.

## Introduction

Coronavirus disease 2019 (COVID-19), a disease caused by Severe Acute Respiratory Syndrome Coronavirus 2 (SARS-CoV-2), was responsible for great efforts by different sectors of society to mitigate and control the impacts caused by the advance and spread of the disease worldwide^1,2^. In at least 2 years since the first report in late 2019^3^, there have been numerous advances with respect to the understanding of the mechanism of action of the virus^4^, forms of transmission^5^, clinical management of infected individuals^6^, diagnostic tests, especially point-of-care testing^5^, the development and availability of safe and effective vaccines in record time^7^, including those based on third-generation technologies^8^, as well as the viral detection in environmental matrices^9^.

Thus, it is understood that these advances have been fundamental for the reduction in the number of cases of COVID-19, as well as decreasing hospitalization and mortality rates of the disease^10^. However, the advance of vaccination has caused a large number of individuals to present with the asymptomatic form of the disease, making clinical diagnosis and, consequently, tracking the advance of the virus more difficult^11^. In addition, the possible emergence of new variants of SARS-CoV-2, with properties commonly related to increased transmissibility and immune escape properties, create a scenario of uncertainty still to be found when it comes to the disease^12^.

Within this perspective, wastewater-based epidemiology (WBE) becomes an interesting epidemiological approach to monitoring the prevalence of SARS-CoV-2, since wastewater constitutes a vast source of biological, biochemical, behavioral and socioeconomic information of a given population^13^. Specifically, WBE presents the possibility for the detection of new cases of the disease, since through it there can be a detection of symptomatic and asymptomatic individuals since the latter are often not quantified by the limitations presented by in vitro and clinical tests^13,14^. Thus, WBE can be an important alternative to epidemiology, since traditional methods have important biases and/or limitations, such as spatial coverage of a given location, diagnostic test supply capacity, level of demand at health units, and the very difficult in tracking individuals who have had contact with an infected subject^15^.

Furthermore, its monitoring is be able to predict up to 7 days in advance the increase or decrease in SARS-CoV-2 infections recorded in health centres^16^. Hence, WBE may support the adoption of more effective public health policies by the authorities^17^. Due to these properties, WBE studies have already been carried out in different cities around the world, as well as in small size localities, such as campuses and buildings, and presented data relevant for epidemiological surveillance so that there was a compression of spatial and temporal trends in the circulation of SARS-CoV-2 in that particular population^18–20^.

In Brazil, monitoring has been carried out since the beginning of 2021 in some of the main cities in the country, such as Belo Horizonte, Curitiba, Distrito Federal, Fortaleza, Recife and Rio de Janeiro, whereby in the epidemiological bulletins issued up to March 2022, the monitoring data showed a correlation between trends of increase and decrease in the amount of SARS-CoV-2 genetic material present in sewage and the numbers of suspected and confirmed cases of COVID-19 in each city evaluated^21,22^. Recently, sewage monitoring data from the cities above not only showed the increase in infections caused by the omicron variant but also anticipated between 7 and 15 days the increase in the number of new cases^23^. However, to the best of our knowledge, no WBE monitoring for SARS-CoV-2 has been carried out in Salvador city, the fourth most populous city in the country and the largest in Brazilian Northeast^24^. During the pandemic of COVID-19, Salvador presented an important role from the epidemiological point of view, being the city with the highest number of confirmed cases in the state of Bahia and the second highest in the Northeast region^25^. At different moments of the pandemic, the epidemiological profile of the reported cases of COVID-19 in Salvador showed an upward trend when compared to data from Brazil, indicating the relevance of studying the epidemiological behaviors of this municipality^26^.

Further, the accurate COVID-19 WBE monitoring possess some challenges to overcome, included the methodologies strategies employed to concentrate, quantify and recovery RNA from samples. The currently methods used for this purpose are still being refined and can ranging regarding the wastewater sampling source and volume chosen (e.g., solid or aqueous portions), different concentration methods (e.g., precipitation, ultrafiltration, adsorption), pretreatments (e.g., different approaches to removal of solids and/or thermal inactivation of wastewater samples) and RT-qPCR approach employed^27,28^. Thus, optimizing and standardizing WBE methodology is fundamental to unify the results from different sources and increasing the sensitivity of the COVID-19 WBE monitoring approach, since different methods can lead to different quantifications of SARS-CoV-2 even in the same wastewater sample studied^29^.

Previously, our group evaluated and validated the efficiency of four methods to concentrate and determine SARS-CoV-2 from wastewater and wastewater-enriched river water samples. We observed that ultrafiltration and PEG 8000 precipitation showed the best SARS-CoV-2 concentration efficiency to wastewater and river water samples, either in aqueous and suspended solids samples or without pretreatment needed for suspended solid removal^30^. Herein we build on our previous work by applying the wastewater surveillance approach, employing PEG 8000 precipitation methodology, to monitor the prevalence of SARS-CoV-2 in raw and treated wastewater samples from 22 municipal wastewater treatment plants (WWTPs) located in Salvador, Bahia State, Brazil.

## Results and discussion

### Successful detection of SARS-CoV-2 in wastewater samples

**Figure 1:**
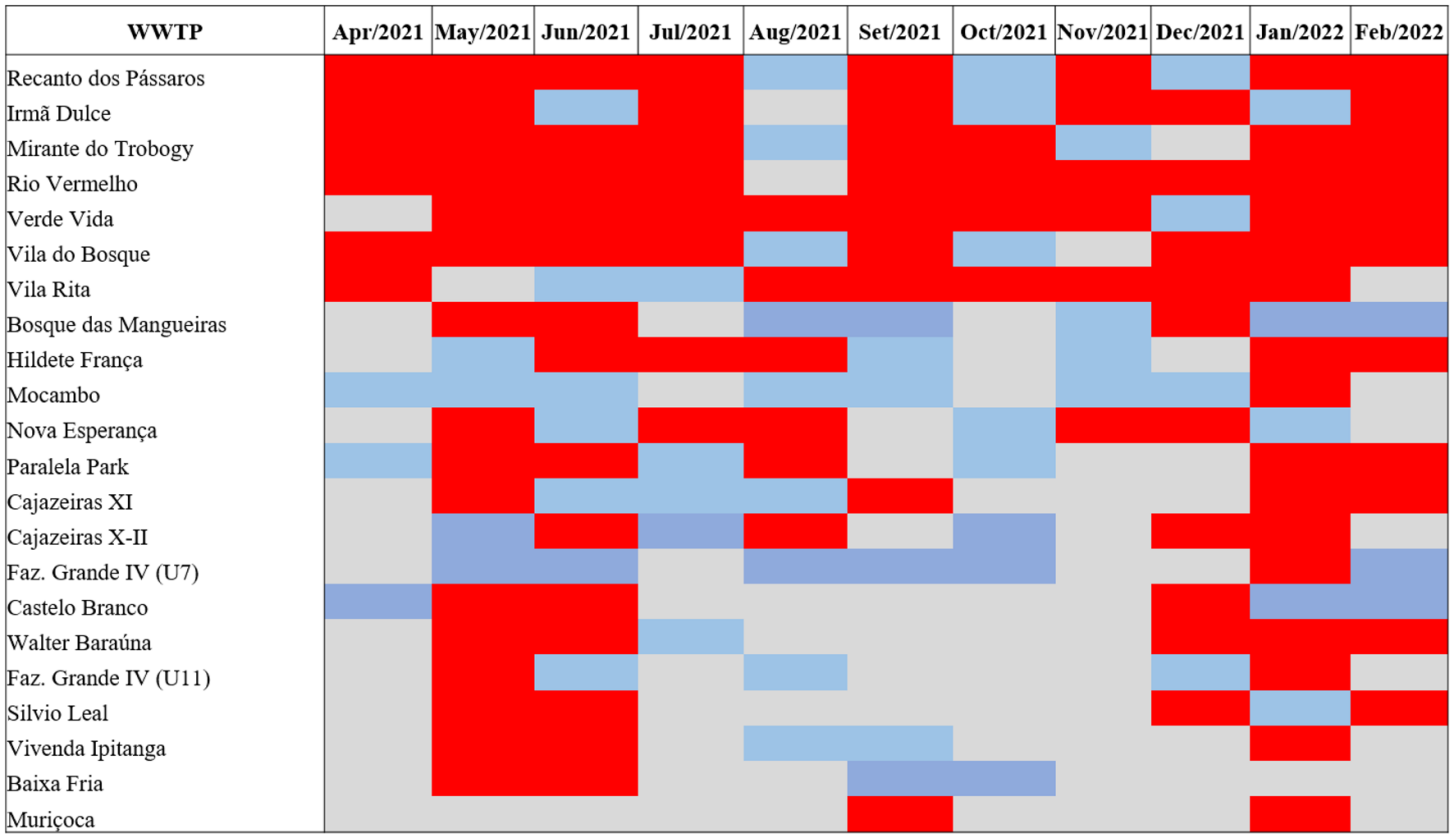
Overview of data analysis for SARS-CoV- 2 detection from samples of different WWTPs in Salvador city. Samples were collected between Apr/2021–Feb/2022 from 22 WWTPs localized in Salvador city. The data are presented as results from any sample positive for SARS-CoV-2 genome, unregarding the employment of N1 or N2 marker, concentration step (PEG 8000), as well as the kind of sample used (raw or treated wastewater). Red marking: WWTPs SARS-CoV-2 positive; blue marking: WWTPs SARS-CoV-2 negative and gray marking: samples not available.

The first step of this study was to demonstrate the success of the SARS-CoV-2 detection methodology in samples from 22 WWTPs of Salvador city, regardless of the sample type analyzed (raw or treated wastewater), the performance of concentration step by PEG 8000 and the employment of N1 or N2 markers. According to Fig. 1, all WWTPs evaluated tested positive for the presence of RNA from the new coronavirus at some point in the long-term monitoring. Besides, the months of May/2021, June/2021 and January/2022 had about > 80% of the evaluated WWTPs, as well as a higher positivity rate (< 65%) for SARS-CoV-2 detection. The number of viral copies (viral load) per reaction of each WWTPs/month is presented in Supplementary Table S1, considering reactions by N1 marker. As in the Prado et al.^17^ study, the presence of SARS-CoV-2 genetic material was detected in sewage samples throughout the follow-up period of between April and August 2020 in the city of Niterói, a Brazilian city in the state of Rio de Janeiro with an estimated population of more than 500,000^31^.

Importantly, among all WWTPs evaluated, only Rio Vermelho remained positive in all the evaluated months, representing a powerful candidate for monitoring the presence of SARS-CoV-2 in wastewater, since receives about 70% of Salvador's sewage^32^. Data from EMBASA show that the referred WWTP has a pre-conditioning station with a processing capacity of 8.3 thousand liters per second, and that it operates in dry weather (when it is not raining) with 7.5 thousand liters of effluents per second, which gives an idea of its dimension and importance for the city^33^. Additionally, it is important to highlight that expand the city's sanitation network, EMBASA has encouraged the installation of WWTPs inside residential condominiums, resulting in decentralization and expansion of sewage collection ^34^.

Different authors have demonstrated the success of monitoring wastewater coming from smaller locations, such as university campus or residential condominiums, highlighting the possibility of adopting measures to contain SARS-CoV-2 transmission considering these locations as well^20,35^. Twenty-one WWTPs analyzed in this study are within this perspective (except for Rio Vermelho WWTP), which makes their inclusion interesting for the analysis of WBE in Salvador.

### Alternatives for the detection of SARS-CoV-2 in different types of wastewater samples

Next, raw wastewater and treated wastewater samples were used to determinate the influence of sample type and concentration step on SARS-CoV-2 positivity rate of WWTPs over time. For this purpose, analyses performed with the N1 marker were considered for the analysis and discussion in the main text of the paper due to its higher sensitivity compared to other SARS-CoV-2 molecular markers, including the N2 marker^14,36^. However, data obtained using the N2 marker are also presented in the supplementary material in Supplementary Figures S1-S3.

**Figure 2:**
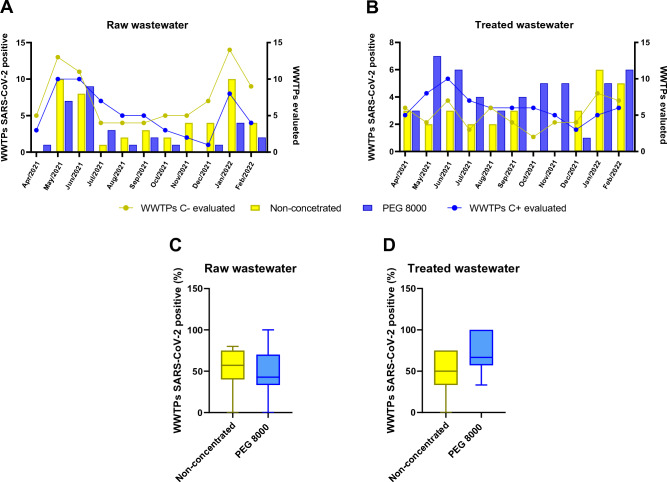
Influence of sample characteristic and concentration procedure step in detection of SARS-CoV-2 from WWTPs of Salvador city. Samples were collected between Apr/2021–Jan/2022 from tween-two WWTPs through Salvador city. Overview of total and positive WWTPs evaluated for SARS-CoV-2 genome for (**A**) raw wastewater and (**B**) treated wastewater samples, submitted or not to PEG 8000 concentrations. The Percent of positive WWTPs for SARS-CoV-2 for either (**C**) raw wastewater or (**D**) treated wastewater samples is presented. The viral genome detection was performed via RT-qPCR using the N1 primer, as described in Methods section. Bars represent mean ± SD values of positivity rate for SARS-CoV-2 of samples from different WWTPs in Salvador. Mann–Whitney non-parametric t-test was used for comparisons between two groups. C −: non-concentrated; C + : PEG 8000 concentration: WWTP: wastewater treatment plant.

**Figure 3:**
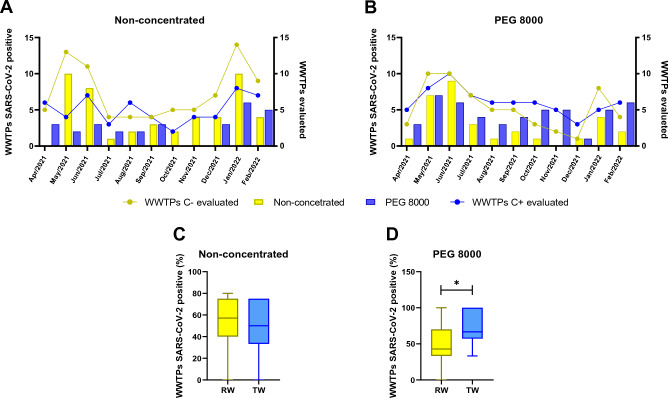
Influence of step concentration procedure in detection of SARS-CoV-2 in raw and treated wastewater samples. Samples were collected between Apr/2021–Jan/2022 from tween-two WWTPs through Salvador city. Overview of total and positive WWTPs evaluated for SARS-CoV-2 genome in raw wastewater and treated wastewater samples (**A**) non-concentrated or (**B**) PEG 8000 concentrated. Percent of positive WWTPs for SARS-CoV-2 for either (**C**) non-concentrated and (**D**) PEG 8000 concentrated wastewater samples is presented. The viral genome detection was performed via RT-qPCR using the N1 primer, as described in Methods section. Bars represent mean ± SD values of positivity rate for SARS-CoV-2 of samples from different WWTPs in Salvador. Mann–Whitney non-parametric t-test was used for comparisons between two groups (*p < 0.05). C −: non-concentrated; C + : PEG 8000 concentration: WWTP: wastewater treatment plant.

In general, the genetic material of SARS-CoV-2 could be detected in both raw and treated samples during the monitoring period (Fig. 2), as well as in samples that did or did not undergo the concentration step with PEG 8000 (Fig. 3). Thus, regardless of the sample type or whether or not the concentration method (use of PEG 8000) was present, SARS-CoV-2 was identified at some analysis point during the monitoring period. The number of positive samples, represented by the number of WWTPs positive for SARS-CoV-2, presented higher detection rates mainly in May and June/2021 months, as well as January/2022 month (Figs. 2 and 3). However, it is noted that the presence of SARS-CoV-2 was not detected in raw non-concentrated wastewater samples in April/2021, while the previously concentrated PEG 8000 raw sample from a WWTP collected in the same month was positive (Figs. 2A and 3A). None of the WWTPs that had the raw samples concentrated with PEG 8000 collected in the month of November/2021 tested positive for SARS-CoV-2 (Figs. 2A and 3B).

Regarding treated wastewater samples, only non-concentrated treated wastewater samples presented negative results for SARS-CoV-2 in both October/2021 and November/2021 months, even though non-concentrated raw samples from several WWTPs for the same period showed the presence of SARS-CoV-2 in the collected material (Figs. 2B and 3A). The results suggest that raw or treated wastewater samples have an overall similar positivity rate for SARS-CoV-2, regardless of the usage of PEG 8000 concentration step (Fig. 2C and D). On the other hand, PEG 8000 concentration step appears to increase the positivity rate for RNA virus in treated wastewater samples compared to raw wastewater samples (*p* < 0.05) (Fig. 3D). Similar results were obtained using the N2 marker, where no detection of viral genetic material was observed in raw PEG 8000 concentrated or non-concentrated samples in April/2021 or November/2021, respectively; whereas in none of the treated wastewater samples, regardless of the concentration method employment, was it possible to detect SARS-CoV-2 in October/2021 and December/2021 (Supplementary Figures S1A and S1B). Differently from the samples analyzed employing N1 marker, no statistical difference was observed in the positivity rate for viral RNA in the samples evaluated using N2 marker (Supplementary Figures S1C-1D and S2C-D).

The adoption of different concentration methods to enable viral detection, including SARS-CoV-2, in residual water samples has gained considerable notoriety in recent years, especially during the COVID-19 pandemic, where WBE has become extremely relevant. The apparent instability of the RNA molecule due to possible interactions with organic materials or another chemical substance^37,38^, widely available, is widely available in raw sewage samples and composition implies poor viral recovery, detection, and reproducibility^39^. These issues have resulted in numerous efforts being directed towards elucidative methods that can mitigate this scenario, allowing analysis of the presence of the virus in wastewater to become a viable tool. Within this perspective, different authors have directed their work to compare different concentration methods, aiming to analyze the sensitivity for the recovery of SARS-CoV-2 in wastewater^40–42^.

The recovery of the genetic material of SARS-CoV-2 by concentration using PEG 8000 (which is based on the precipitation analysis) has shown a better yield when compared to other techniques, such as flocculation or ultracentrifugation^30,43,44^. Therefore, a lower concentration of organic matter (or reaction inhibitors) in the treated samples, as well as the application of a concentration technique capable of increasing the efficiencies of viral recovery may explain the superior performance of the positivity rate of the treated samples that were concentrated by PEG 8000 compared to the others analyzed in this study. An important highlight is simplicity of the PEG-based concentration technique since there is no need for specialized or high-cost equipment such as centrifugation ^45^. This feature is of great importance in times of crisis and in places where financial resources for the development of research are limited, as is the context of Brazil.

### Long-term monitoring of SARS-CoV-2 in wastewater samples

**Figure 4:**
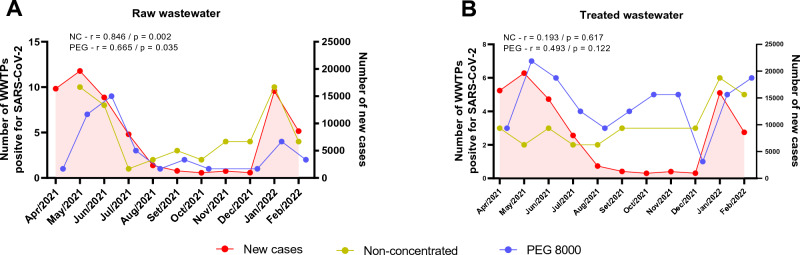
Relationship between average new cases of COVID-19 and SARS-CoV-2 detection in samples from different WWTPs of Salvador city. (**A**) Raw wastewater and (**B**) treated wastewater samples were collected between Apr/2021–Jan/2022 and submitted or not to PEG 8000 concentration. SARS-CoV-2 RNA was detected via RT-qPCR using the N1 primer, as described in Methods section. The correlations between non-concentrated or PEG 8000 concentrated raw wastewater with the number of new cases for COVID-19 and between non-concentrated or PEG 8000 concentrated treated wastewater with the number of new cases for COVID-19 are shown in each graph (**A**) and (**B**), respectively. Pearson test was used to verify the correlations. The r and *p* values are plotted in each graph.

Monitoring for the presence of SARS-CoV-2 in the period April/2021 to February/2022 was also conducted in this study. The WBE data presented high detection of SARS-CoV-2 in the months of May and June/2021 months, as well as January/2022 month, which coincide with the increase in new cases of coronavirus infection registered in the city (Fig. 4). It is noted that the decline in the number of cases and, consequently, the number of positive WWTPs observed from April/2021 onwards may be associated with the end of the second wave of COVID-19 cases in Brazil, characterized mainly by cases of infection or reinfection by the Gamma (P.1 lineage) variant of SARS-CoV-2^46–48^. Although it was first identified in the Amazonas state, northern Brazil, causing increased morbidity and mortality from COVID-19^49^, the study Nonaka et al.^50^ showed that the genome of the Gamma variant was also sequenced from samples of patients hospitalized in intensive care units in Salvador, thus having an important epidemiological role in the city.

On the other hand, the increase in the number of new cases and positive WWTPs observed from December 2021, with a considerable peak in January/2022, may be related to the global spread of the Omicron variant of concern (B.1.1.529 lineage), since about 95% of the SARS-CoV-2 genomes sequenced in private health services and Brazilian cities belong to this variant in this period^51^. As reported in other countries around the world^52,53^, soon after the first case with Omicron in Brazil, the spread of the variant was reported in all regions of the country, increasing the number of cases and deaths, especially during the month of January/2022^54,55^. However, despite reaching the peak of new cases in a rapid manner, the rapid decline in the number of cases was also reported^55^. This behavior was also similar to that found in Fig. 4 between January and February/2022, where there was a reduction in the number of new cases and positive WWTPs.

Thus, in general, the analysis of Fig. 4 reinforces the sensitivity of wastewater epidemiology as a complementary method to the epidemiological monitoring of SARS-CoV-2, since it shows that correlations exist between the number of WWTPs positive for SARS-CoV-2, regardless of sample type and analysis methods (with or without concentration by PEG 8000).

Although both non-concentrated and PEG 8000 concentrated raw wastewater samples were able to demonstrate the presence of SARS-CoV-2 RNA over time, there is a strong positive correlation between non-concentrated raw wastewater samples and the number of new cases for COVID-19 (r = 0.846, *p* = 0.002), as well as to raw non-concentrated sample evaluated using N2 marker (r = 0.709 and *p* = 0.014) (Supplementary Figure S3A), suggesting the ability of WBE based in this sample type in predicting new cases of COVID-19 disease. The detection of SARS-CoV-2 genetic material in raw and non-concentrated samples, especially during periods of a high number of new cases, demonstrates the possibility of employing a simple and direct analysis using sewage samples without the viral particle concentration step. This could be an advantage for poor countries by allowing an efficient and low-cost WBE method for monitoring the prevalence of SARS-CoV-2.

However, it is important to show that, despite the low correlation between the new number of cases and the viral load of the treated and concentrated samples (PEG 800) (r = 0.493, *p* = 0.122), it is noted that it presented a higher positivity rate over time. This scenario may be related to the low notification (underreporting) rate of COVID-19 cases found in Brazil, as well as countries such as Iran, Spain, France, the United States, and Mexico^56,57^. Prado et al.^17^ showed that the number of cases of COVID-19 in Brazil can be up to 11 times higher, and the notification rate for all the national states was less than 30%. The study of Kupek^58^ showed that by the year 2020, the number of underreporting of COVID-19 deaths in Brazil could reach 22.62%.

The imminent emergence of new Omicron subvariants, such as BA.5, XBB, BQ.1 and, more recently, BQ.1.1, have increased the uncertainty surrounding the control of the pandemic worldwide, even though none of them is globally dominating the number of new cases^59^. The new variants have presented advantages related to immune escape due to mutations in the SARS-CoV-2 receptor-binding domain, the region responsible for virus entry into the host cell, and the main target of vaccines or diagnostic kits^60,61^. These modifications may result in the need for the adoption of new public policies directed at confronting the spread of COVID-19^62^, which makes a scenario of underreporting of new cases or deaths a limiting factor for the effectiveness of these actions. Moreover, even with the approval of Omicron variant vaccines, there is a lack of equity in the availability of these products globally, which reinforces the need for the adoption of additional tools in these locations, as is the case in Brazil^63^.

Therefore, the use of WBE as a complementary tool in times of uncertainties like the one mentioned becomes fundamental, since it can overcome the limitations of establishing mass testing initiatives for the population that is associated with the availability of diagnostic kits and the willingness of the population to go to a health unit to be tested^64^. Although it was not the aim of this study, it is important to point out that WBE can indicate which SARS-CoV-2 variants are circulating in a given locality at a specific period, and can also detect the occurrence of new variants for that same community^65^.

**Figure 5:**
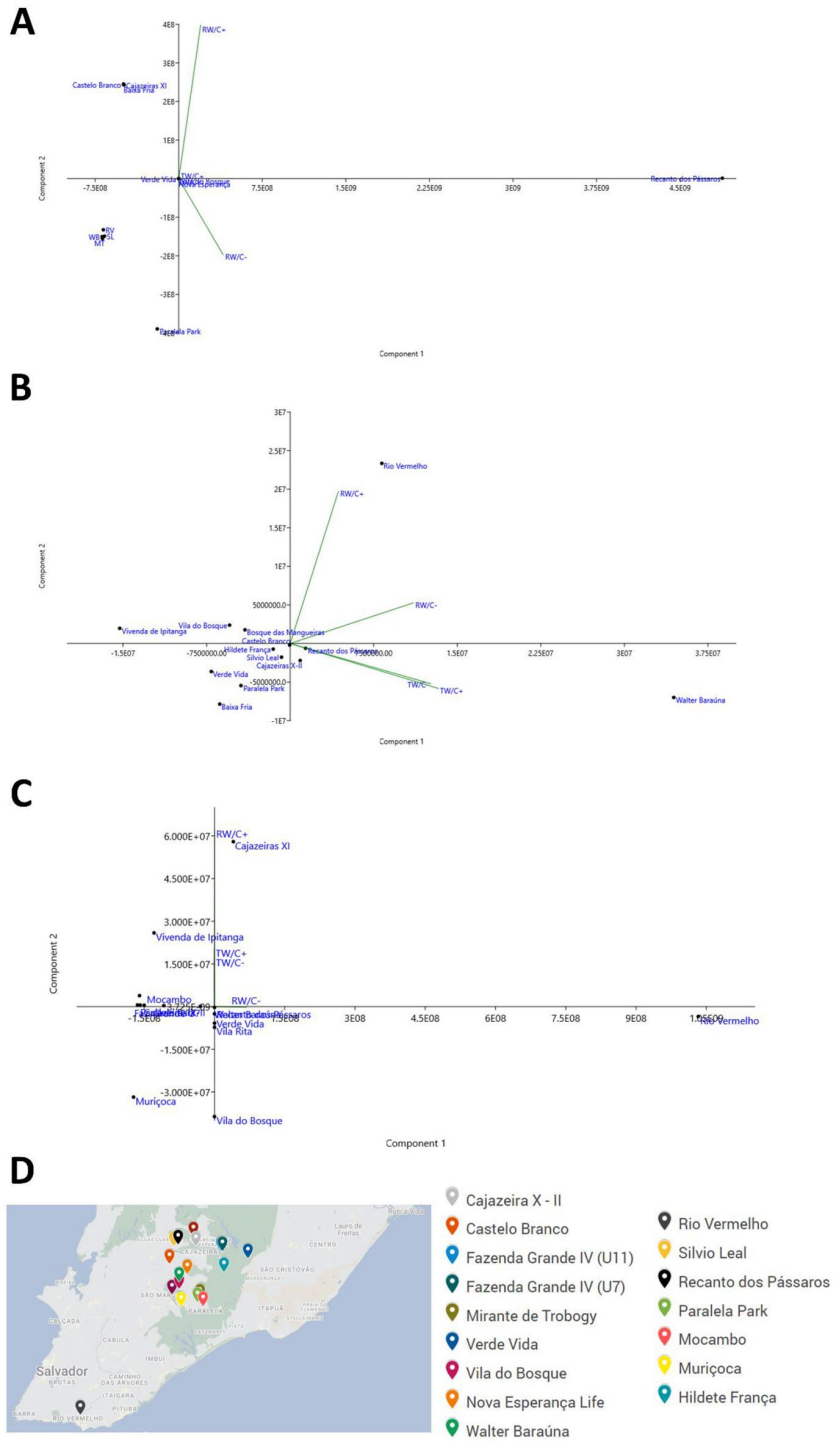
Scatter plot by principal component analysis of viral loads found in TEEs in the months of (**A**) May/2021; (**B**) June/2021 and (**C**) January/2022; (**D**) Represents the geolocation of each analyzed WWTP in these periods.

An important fact demonstrated by Figs. 2, 3 and 4 is that the months of May/2021, June/2021 and January/2022 are the months that had the highest number of positive WWTPs, regardless of the type of sample or the presence or absence of the concentration by PEG 8000. This interpretation is also observed through the number of viral copies (viral load) presented in Supplementary Table S1, where the samples collected and analyzed during the mentioned months present the highest viral loads identified. Given the importance of applying WBE to monitor the spread of SARS-CoV-2 in a given geographical area and considering the large volume of data for the same WWTP (raw wastewater (RW) or treated (TW); concentrated (C +) or not concentrated (C −)), principal component analysis was applied in to assess information about the possible grouping of samples (WWTPs) based on the applied methodology, being able to extract detailed information that would otherwise be lost when using more traditional statistical methods (Fig. 5).

In May/2021, data relating to RW/C − samples (raw wastewater samples that were not concentrated) explained 98.24% of the total variance of the data set, thus becoming principal component 1. Principal component 2 showed a variance of 1.75%, being related to RW/C + (raw wastewater samples that were concentrated). Thus, the first principal components explained 99.99% of the total variance (Fig. 5A). Differently, in June/2021 the highest variances were found for TW/C + (treated wastewater samples that were concentrated) with 60.35% and 26.38% for TW/C- (treated wastewater samples that were not concentrated), resulting in 86.73% of the total variance and, consequently, of the data explanation (Fig. 5B). The month of January/2022 had a variance of 99.9% only for principal component 1, represented by RW/C −, pointing to its relevance (Fig. 5C). Noteworthy is the role of raw wastewater sample variables, concentrated or not, for requiring high loadings in May and June of the year 2021. The geolocation of each analyzed WWTP in the mentioned periods is shown in Fig. 5D.

In general, it was shown that nearby WWTPs tend to form clusters, such as Fazenda Grande U11 and Fazenda Grande U7, indicating a similar viral load (Fig. 5). However, it is important to show that in June/2021 and January/2022 the Rio Vermelho WWTP was not grouped with any other WWTP, which is expected, since it is a WWTP that receives more than 70% of the sewage from the city of Salvador, which may have impacted on a higher viral load compared to other WWTPs with lower capacity. Thus, the results suggest that the location and capacity of the WWTP can interfere with the viral load found in the sample. Wade et al.^66^ point out that one of the main challenges associated with WBE is the variation between geographic locations, as lower catchment WWTPs may have lower viral concentrations than the higher catchment, even though the prevalence of disease may be the same in both catchment regions.

**Figure 6:**
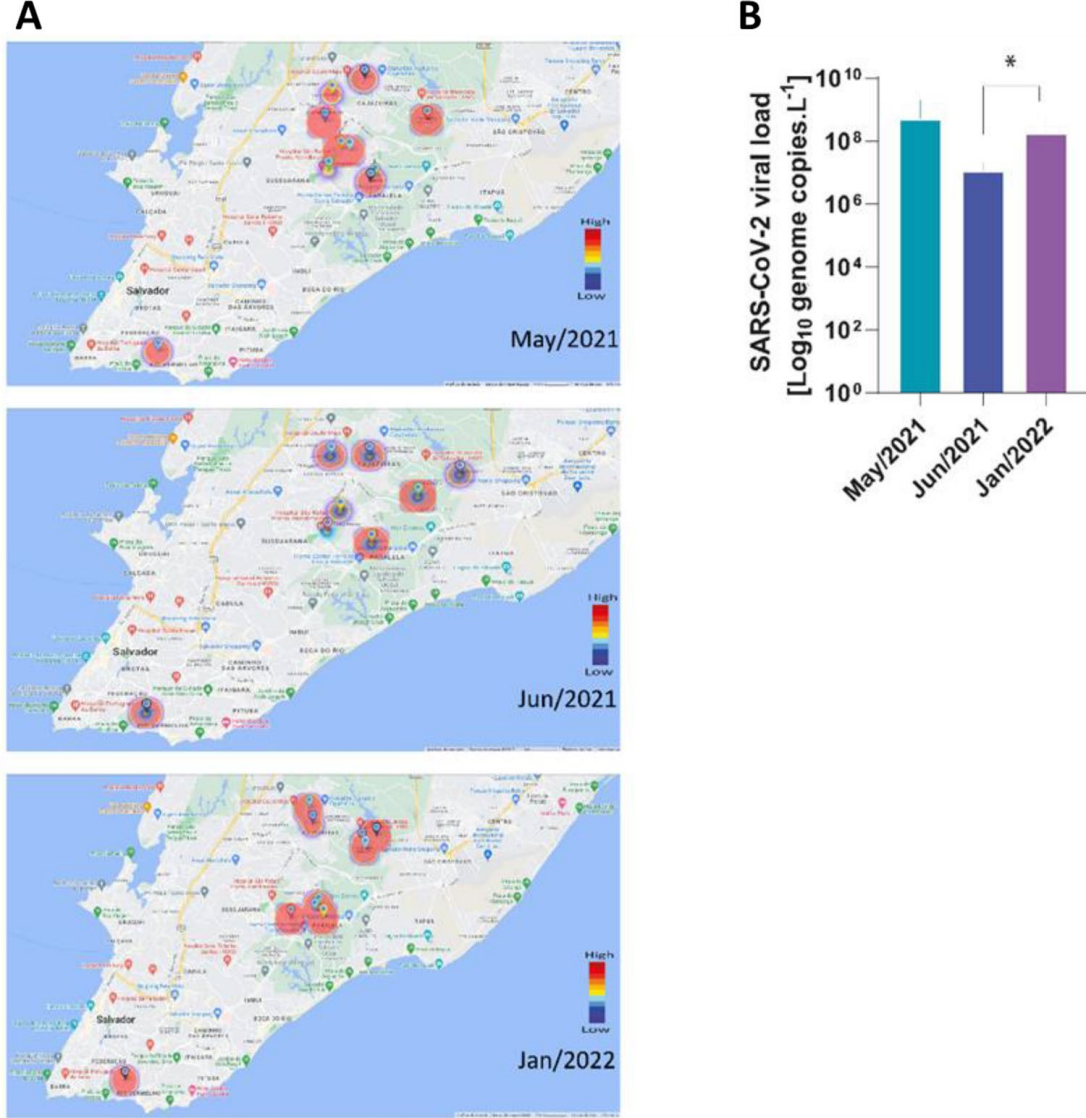
SARS-CoV-2 viral load in non-concentrated raw wastewater samples during high COVID-19 detection periods. Samples were collected between May–June/2021 and January/2022 from different WWTPs localized in Salvador, and the viral genome detection was performed via RT-qPCR using the N1 primer, as described in Methods section. (**A**) Heatmaps show an abundance of SARS-CoV-2 RNA copies in distinct periods. (**B**) SARS-CoV-2 genome copies from samples collected between May–June/2021 and January/2022. Bars represent mean ± SD values of viral copies (Log10) from Jun/2021 and Jan/2022. The Kruskal–Wallis nonparametric test, followed by Dunn's posttest, was used to compare among experimental groups (*p < 0.05).

Given the importance highlighted by the principal component analyses pointed to the raw wastewater samples, Fig. 6 presents the viral load found in the samples from May, June and January according to the TSS in the heat map representation (Fig. 6A). It is noteworthy that significant difference (*p* < 0.05) was found when comparing the total viral load of June and January, suggesting the relevance of the rapid spread of the Omicron variant (Fig. 6B). These data reinforce the perspective on the importance of adopting WBE for monitoring COVID-19.

## Methods

### Raw and treated wastewater samples collection

**Figure 7:**
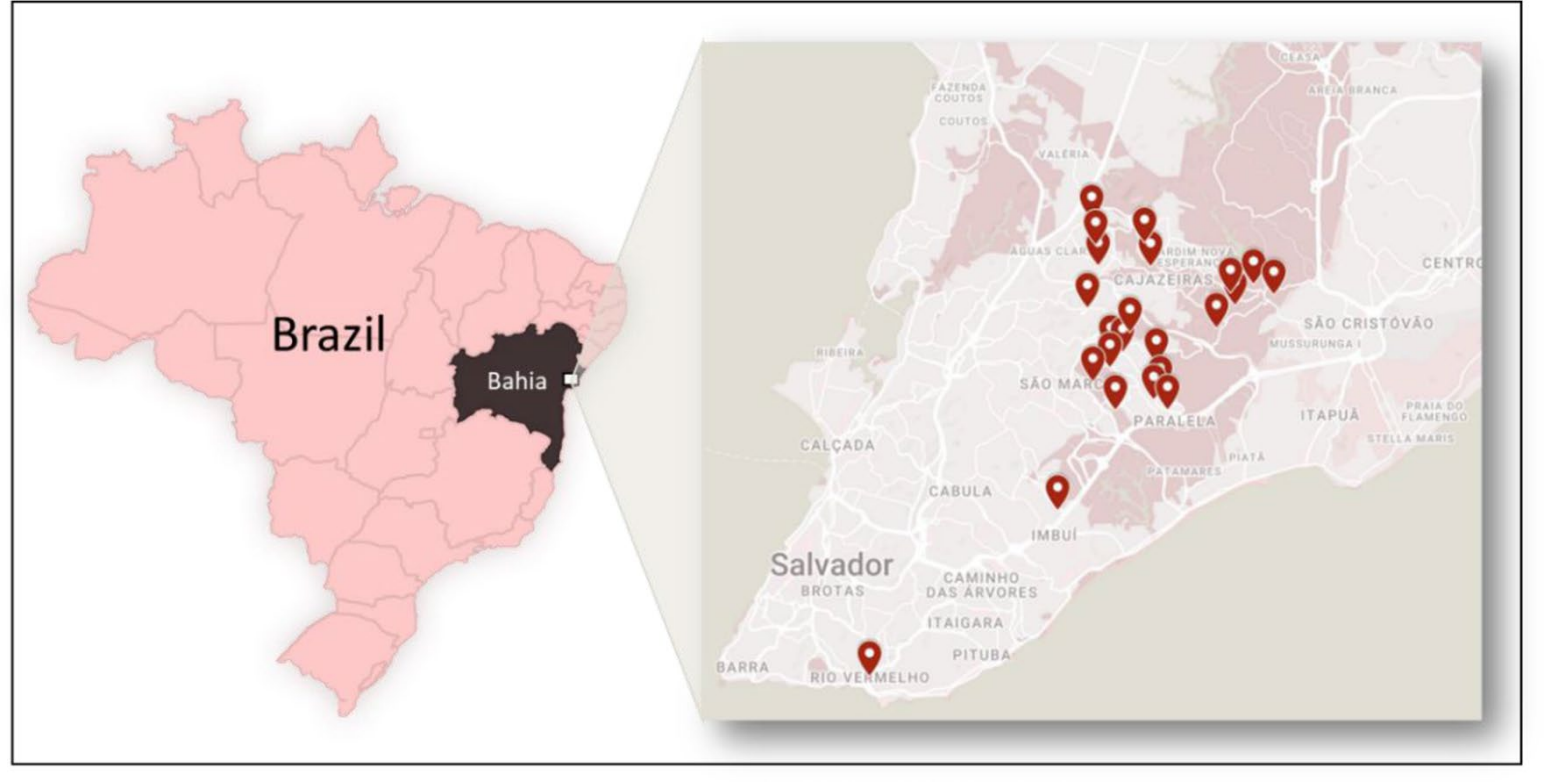
Spatial distribution of the WWTPs evaluated. Samples were collected between Apr/2021–Feb/2022 from 22 WWTPs localized in Salvador, Bahia, Brazil.

Raw and treated wastewater samples were provided by the collaborators of Salvador Municipality Effluent Treatment Company, Empresa Baiana de Águas e Saneamento (EMBASA) from 22 different wastewater treatment plants (WWTPs) within Salvador, Bahia, Brazil, between April/2021–February/2022 (Fig. 7).

**Figure 8:**
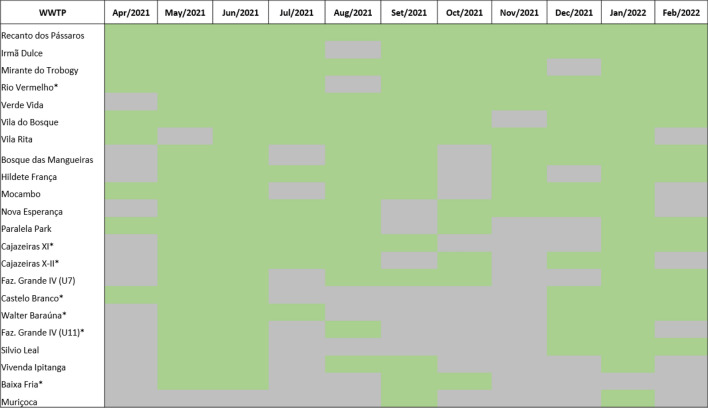
Overview of sample collection per WWTP. Raw and treated wastewater samples were collected between Apr/2021–Feb/2022 from 22 WWTPs localized in Salvador city. *: WWTPs that possess only raw wastewater sample collected; green marking: sample collected and gray marking: samples not available.

Raw and treated wastewater samples were collected using 0.5 L polypropylene flasks directly from the collection tank of the treatment plant. The homogenized samples were conditioned and transported at 4 ± 2 °C and then stored at − 80 °C. To increase the biosafety for sample manipulation, a thermal inactivation protocol was performed as described by Binivis et al.^67^ and Wu et al.^68^. The samples were maintained at low temperatures (-80 ± 2 °C) for further analyses. The methodology used for sample concentration, RNA extraction, and quantification is described in the following sections. Identification of WWTP, what kind of samples were collected and how long the monitoring are shown in Fig. 8.

### Experimental design and controls

All samples of this study were evaluated in two experimental flows: (1) without a step for viral particle concentration (C −) or (2) using a precipitation protocol to obtain a 40-fold concentrated sample (C +). For each sample experimental flow analyzed (C − or C +), duplicates were performed to obtain two independent RNA extractions for RT-qPCR runs. All RNA extraction was performed in duplicate in the RT-qPCR assays.

In order to evaluate the performance of the RNA concentration and extraction steps, in addition, to determining the eventual occurrence of inhibition in RT-qPCR experiments, lentiviral particles containing a plasmid with a sequence for a green fluorescent protein (GFP) were used as control. Lentiviruses are enveloped viruses members of the Retroviradae family, commonly used in biopharmaceutical studies. The GFP-lentivirus was obtained from mammal cell cultures, quantified and then added at a concentration of 5 × 10^5^ gc/µL to 40 mL of C + samples, before PEG concentration step (details in supplementary data). For C- samples, this GFP-lentivirus control (5 × 10^5^ gc/µL) was added into 1 mL of each sample in parallel with C + samples preparation for PEG concentration procedure. Thus, the evaluation of the recovery efficiency using this external control could be performed individually by sample and then a comparison of C − and C + samples on lentivirus recovery could be performed to evaluate the eventual viral loss during concentration. To calculate the number of gene copies per reaction, the equations obtained from the Standard Curves (N-CoV and eGFP) are used. The equation is (elution buffer volume related to RNA x copy number)/volume of concentration, and it was described in a technical note from INCT^69^.

### Wastewater concentration and RNA extraction

Polyethylene glycol (PEG) precipitation method and RNA extraction were performed as reported previously^30^. To PEG precipitation method, PEG 8000 (10% w/v) and sodium chloride (NaCl) (2% w/v) were added into 40 mL of sample and homogenized until completely dissolution of the powders. The samples PEG/NaCl-added were incubated overnight at 4 °C under agitation. Afterwards, samples were centrifuged at 4863 × *g* (maximum centrifuge speed) for 60 min at 4 °C. Supernatants were discarded and the precipitated fraction, which contains the viral particles, was resuspended in PBS 0.01 M solution (pH 7.2) until reach 1 mL as the final sample volume. RNA extraction was performed using the MagMax™ Viral/Pathogen II kit (Thermo Fisher Scientific™, Massachusetts, United States) in a semi-automated system KingFisher™ Duo Purification System (Thermo Fisher Scientific™, Massachusetts, United States) following the manufacturer’s instructions. Immediately after RNA extraction, the obtained nucleic acids were submitted to PCR inhibitors removal by using the OneStep PCR Inhibitor Removal Kit (Zymo Research, California, United States). Finally, the cleaned RNAs where adjusted to 100 µL with the elution buffer provided within MagMax™ Viral/Pathogen II kit. Concentrated samples and extracted/cleaned RNAs were stored at − 80 ± 2 °C until analysis.

### RT-qPCR assay

Detection of SARS-CoV-2 RNA in samples was performed using genetic targets N1 and N2 located in SARS-CoV-2 nucleocapsid gene, and those primers were evaluated by the Center for Disease Control and Prevention ^70^. For eGFP-lentivirus control detection, primers and probes were designed by the authors for this study using the software PrimerQuest™ Tool (IDT, California, EUA) applying the default settings. Of all sequences provided by the PrimerQuest™ Tool, assay set #1 constituted of forward primer, 5′-GAACCGCATCGAGCTGAA-3′; reverse primer, 5′-TGCTTGTCGGCCATGATATAG-3' and the probe sequence, 5′-6-FAM—ATCGACTTCAAGGAGGACGGCAAC—BQ-1 -3′. The evaluation of a combination of several eGFP primers and probes concentrations were tested and the concentrations of 400 nM for each, forward and reverse eGFP primer, and 200 nM for eGFP probes were selected for this study. Details about primer and probe testing are available in supplementary data.

The RT-qPCR assay, iTaq Universal Probes One-Step RT-qPCR Kit (Bio-Rad, California, United States) were used according to the manufacturer’s instructions. Reactions were performed for N1 and N2 targets with 5 μL of the extracted and cleaned RNA, 10 µL of 2 × iTaq PCR reaction mix, 1.5 µL of SARS-CoV-2 RUO qPCR Primer & Probe Kit (IDT, California, EUA) (N1 and N2), 0.5 µL of iScript reverse transcriptase and 3 µL of nuclease-free water (NFW) reaching a final volume of 20 µL. A standard curve with five tenfold serial dilution points (10^6^ to 10^1^) using 2019-nCoV positive control plasmid (IDT, California, EUA) was performed in RT-qPCR assays and also employed as positive control. Respecting to eGFP RT-qPCR assays, reaction components volumes were 5 μL of RNA sample, 10 µL of 2 × iTaq PCR reaction mix, 1 µL of each primer (400 nM each) and probe (200 nM), totalizing 3 µL, 0.5 µL of iScript reverse transcriptase and 1.5 µL of NFW, also totalizing a 20 µL of final volume. A standard curve for eGFP was made from five tenfold serial dilutions (10^6^ to 10^1^) of pEGIP (Addgene plasmid # 26,777; http://n2t.net/addgene:26777; RRID:Addgene_26777), the same used on lentivirus cloning assay, being also used as a positive control. The negative controls were employed though the concentration, extraction and qPCR steps using nuclease-free water following the same procedure as samples. Positive and negative controls were included for each RT-qPCR run for N1, N2 and eGFP molecular targets. All RNA samples were performed in duplicate for the N1, N2 and GFP targets. All assays were performed in this study in 96-wells plate (Ultraflux, SSI) and the amplification reaction occurred in QuantStudio™ 5 Real-Time PCR System (Thermo Fisher).

### Data analyses

Each sample were analyzed individually for the presence or absence of SARS-CoV-2 RNA. Standard curve and threshold data were evaluated in QuantStudio™ Design and Analysis Software v1.5.1 (Thermo Fisher Scientific™, Massachusetts, USA). Cq, slope, intercept, threshold and EAMP data were organized in Microsoft Office Excel™ software (Microsoft Corporation, Washington, USA). The theoretical limit of detection (LOD) of 3 genomic copies per reaction was considered. Only samples with Ct < 40 were validated as positive and Ct values higher than 40 Ct or with absence of amplification were reported as Not Detected (ND). First, the negative controls were analyzed, then the positive controls and, lastly, the standard curve. Data analysis was performed following the MIQE Guidelines developed by Bustin et al.^71^, such as Y-intercept between 33 and 37, R2 ≥ 0.99, and slope values =  − 3.3.

For each sample of raw or treated wastewater, there were two samples (C + and C −) duplicates in the 96 wells plate, totalizing four samples. Samples presenting ≥ 2 amplification were considered positive, while those presenting ≤ 1 amplification were classified as negative since could represent some contamination by pipetting. The number of new cases for COVID-19 for each evaluated month was obtained from the sum of daily cases available on the webpage of Secretaria Municipal de Saúde of Salvador^72^.

### Statistical analyses

Data are presented as means ± standard deviation (SD) from representative experiments. Comparisons between the two groups were performed using the Mann–Whitney non-parametric test. Pearson test was used to verify the significance of the correlation tests. Analyses were performed using GraphPad Prism v.9.00 for Windows (GraphPad Software, San Diego California). The software GraphPad was used as of obtaining the annual GraphPad Prism Group Academic Yearly Subscription license. Results were considered statistically significant when *p* < 0.05. For principal component analysis, the viral loads from the WWTPs were used according to four loads: concentrated or non-concentrated raw wastewater sample, as well as concentrated and non-concentrated treated wastewater). The analysis was performed in PAST (Paleontological Statistics; Oslo, Norway) version 3.26, developed by Øyvind Hammer. Longitude and latitude measurements were employed to build a map of Salvador city containing the spatial distribution evaluated WWTPs in My maps application (Google).

## Conclusion

WBE is an interesting epidemiological surveillance tool that needs methodological refinement and standardization to become an early warning system for SARS-CoV-2. In this context, several studies have been conducted in order to improve the detection of epidemiological targets in wastewater samples. In the present work, we demonstrate the potential of the WBE approach to monitoring the presence of SARS-CoV-2 both locally, in residential buildings, and widely throughout Salvador city. In addition, the SARS-CoV-2 was successfully detected in both raw wastewater and treated wastewater samples and in either PEG 8000 concentrate or non-concentrate samples. The results found in this study reinforce the importance of WBE and its potential usage as a low-cost tool to SARS-CoV-2 surveillance.

### Supplementary Information

Supplementary Information.

## Data Availability

All the results found are available in this manuscript.
