# Supplementary material for: Long-term monitoring of COVID-19 prevalence in raw and treated wastewater in Salvador, the largest capital of the Brazilian Northeast
Source: Sci Rep. 2023 Sep 14;13:15238. doi: 10.1038/s41598-023-41060-1 (PMC10502096; doi:10.1038/s41598-023-41060-1)
Supplement: Supplementary file 1 — Supplementary Information. [file 41598_2023_41060_MOESM1_ESM.pdf]

Supporting information for

Long-term monitoring of COVID 19 prevalence in raw and treated wastewater in Salvador, a largest capital of Brazilian Northeast

Authors: Carolina Araújo Rolo [1], Bruna Aparecida Souza Machado [1,2], Matheus Carmo [1], Rosângela Fernandes [1], Maísa Santos Fonseca [1], Katharine Valéria Saraiva Hodel [1], Jéssica Rebouças Silva [1], Danielle Devequi Gomes Nunes [1], Edna dos Santos Almeida [2], Jailson Bittencourt de Andrade [1,2,3*]

Affiliations:

[1] SENAI CIMATEC, SENAI Institute of Innovation (ISI) in Health Advanced Systems (CIMATEC ISI SAS), University Center SENAI/CIMATEC, Salvador 41650-010, Brazil
[2] SENAI CIMATEC, Manufacturing and Technology Integrated Campus, University Center SENAI CIMATEC, Salvador 41650-010, Brazil
[3] Centro Interdisciplinar de Energia e Ambiente – CIEnAm, Federal University of Bahia, Salvador 40170-115, Brazil

* Corresponding author: E-mail address: jailsondeandrade@fieb.org.br (J. B. Andrade)

1. **Materials and methods**

GFP Primers and probe concentration choice

RT-qPCR assays for GFP quantification were performed using primers and probe designed by the authors using PrimerQuest™ Tool (IDT, Califórnia, EUA) software with standard settings. Reverse primer (5'-TGCTTGTCGGCCATGATATAG-3'), forward primer (5'-GAACCGCATCGAGCTGAA-3') and probe (5′-6-FAM - ATCGACTTCAAGGAGGACGGCAAC - BQ-1 -3′) were resuspend with Tris-EDTA 1X buffer. Different combinations of primers and probe concentration were tested prior to reach the best efficiency for the qPCR reaction. pEGIP plasmid (Addgene plasmid # 26777; http://n2t.net/addgene:26777; RRID:Addgene_26777), containing a Green Fluorescent Protein (GFP) sequence, were used as positive control for qPCR reactions validation. Reverse and forward primers were tested in 4 concentrations; 200nM, 400nM,600nM and 800nM; and; the probe evaluated in 3 concentrations; 150nM, 200nM and 250nM. These primers and probe concentrations were tested in all possible combinations. Reactions were performed using the iTaq Universal Probes One-Step RT-qPCR Kit (Bio-Rad, California, United States) following the manufacturer's protocol. Briefly, for each reaction were used 10 µL of 2x iTaq Reaction Mix, 1 µL of each primer and probe, totalizing 3 µL, 0.5 µL of iScript reverse transcriptase, 1.5 µL of NFW, and 5 uL of pEGIP plasmid used as template, totalizing a 20 µL of reaction volume. pEGIP plasmid was used in 5 concentrations from a 10-fold serial dilution initiating in 1 x 106 copies. Each dilution combination was performed in triplicate. Cycling procedure were performed in QuantStudio 1 Real-Time PCR System (Thermo Fisher Scientific, CA, USA) according to iTaq Universal Probes One-Step RT-qPCR Kit manufacturer's protocol (reverse transcription (50°C, 15min) and initial denaturation (95°C, 2 min), followed by 50 amplification cycles at 95°C for 10 sec, and 60°C for 30 sec). Non-template controls were included in the run. The best calibration curve was reached using 400nM each, for forward and reverse primers, and 200nM for probe concentration with -3.322 slope, 41.754 y-intercept and 0.999 R^2 and 99.993% efficiency. Thus, the efficiency of the reaction was 1,999.

Lentivirus and pGip transfection protocol

To ensure quality control in the assays, lentiviruses (LENTV) transfected with pGip, a plasmid with GFP sequence, were used. LENTV are enveloped viruses from Retroviridae

family which contain genomic RNA that is converted to DNA in the transduced cell by a virally encoded enzyme called reverse transcriptase.

They are species-specific in host range and other viruses have been recognized as pathogens of humans and another mammalian species[1]. In first and second-generation lentiviral vectors cis and trans-acting factors of the Lentivirus are on separate plasmids depending on the viral vector generation. Second-generation lentiviral vectors do not include the accessory virulence factors *vif*, *vpr*, *vpu*, and *nef*[2]. The detailed process was developed by the iPSC Core Facility from Penn Institute for Regenerative Medicine and are available in https://www.med.upenn.edu/ipsccore/documents/lentivirusgeneration.pdf

REFERENCES

1. Durand, S. & Cimarelli, A. The Inside Out of Lentiviral Vectors. *Viruses* **3**, 132–159 (2011).
2. Lentiviral Vectors (1st and 2nd Generation) Biological Agent Reference Sheet (BARS) | Environment, Health and Safety. https://ehs.cornell.edu/research-safety/biosafety-biosecurity/biological-safety-manuals-and-other-documents/bars-other/lentiviral-vectors-1st-and-2nd-generation.

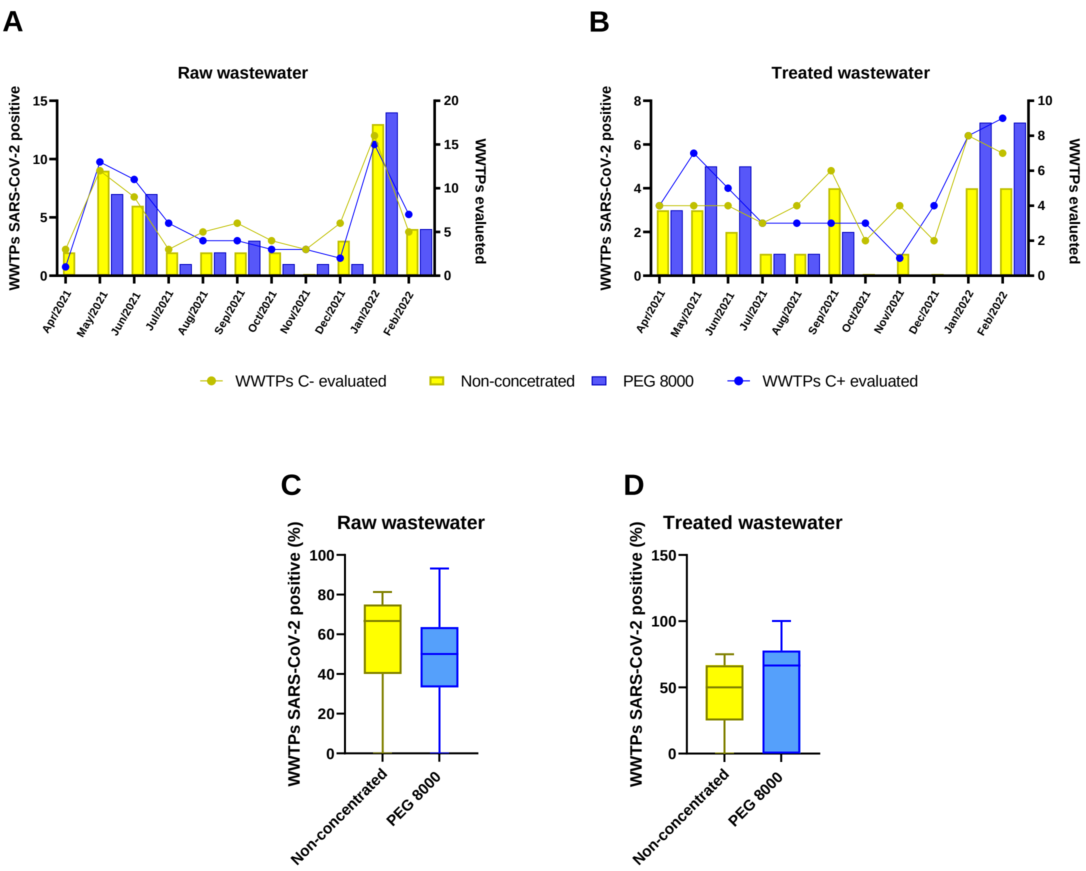

Supplementary Figure S1. Influence of sample characteristic and concentration procedure step in detection of SARS-CoV-2 from WWTPs of Salvador city. Samples were collected between Apr/2021 - Jan/2022 from tween-two WWTPs through Salvador city. Overview of total and positive WWTPs evaluated for SARS-CoV-2 genome for (A) raw wastewater and (B) treated wastewater samples, submitted or not to PEG 8000 concentration. The Percent of positive WWTPs for SARS-CoV-2 for either (C) raw wastewater or (D) treated wastewater samples is presented. The viral genome detection was performed via RT-qPCR using the N2 primer, as described in Methodology section. Bars represent mean ± SD values of positivity rate for SARS-CoV-2 of samples from different WWTPs in Salvador. Mann–Whitney non-parametric t-test was used for comparisons between two groups. C-: non-concentrated; C+: PEG 8000 concentration: WWTP: wastewater treatment plant.

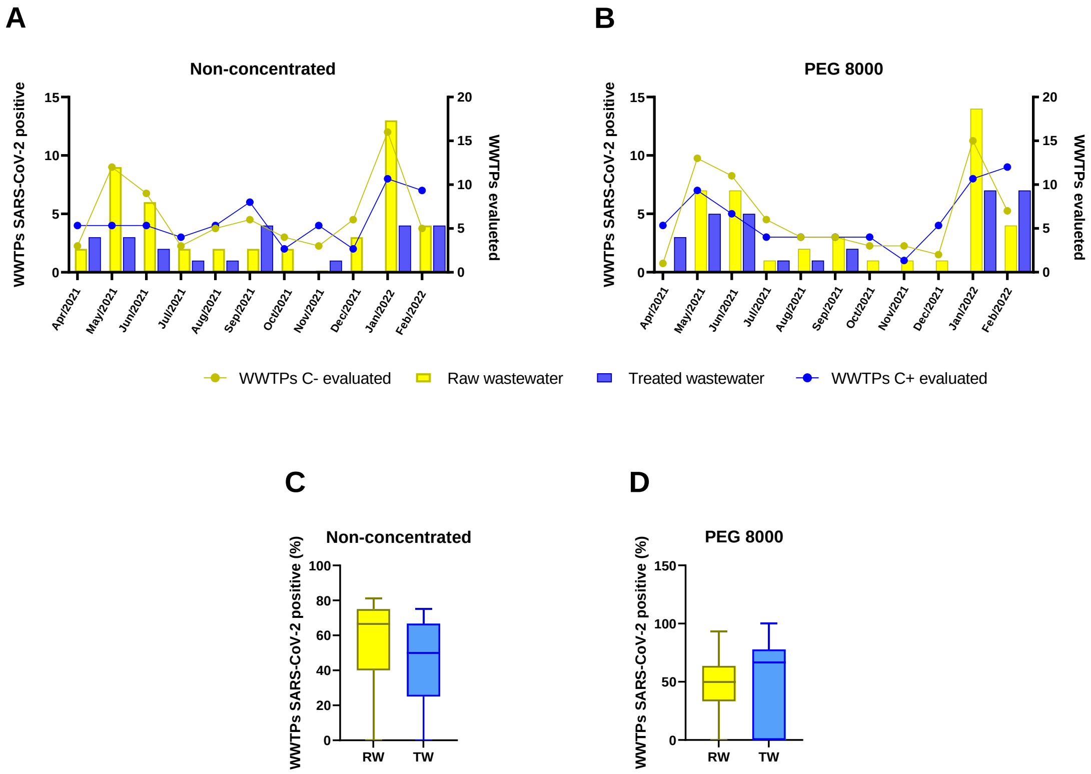

Supplementary Figure S2. Influence of step concentration procedure in detection of SARS-CoV-2 in raw and treated wastewater samples. Samples were collected between Apr/2021 - Jan/2022 from tween-two WWTPs through Salvador city. Overview of total and positive WWTPs evaluated for SARS-CoV-2 genome in raw wastewater and treated wastewater samples (A) non-concentrated or (B) PEG 8000 concentrated. Percent of positive WWTPs for SARS-CoV-2 for either (C) non-concentrated and (D) PEG 8000 concentrated wastewater samples is presented. The viral genome detection was performed via RT-qPCR using the N2 primer, as described in Methodology section. Bars represent mean ± SD values of positivity rate for SARS-CoV-2 of samples from different WWTPs in Salvador. Mann–Whitney non-parametric t-test was used for comparisons between two groups (*p< 0.05). C-: non-concentrated; C+: PEG 8000 concentration: WWTP: wastewater treatment plant.

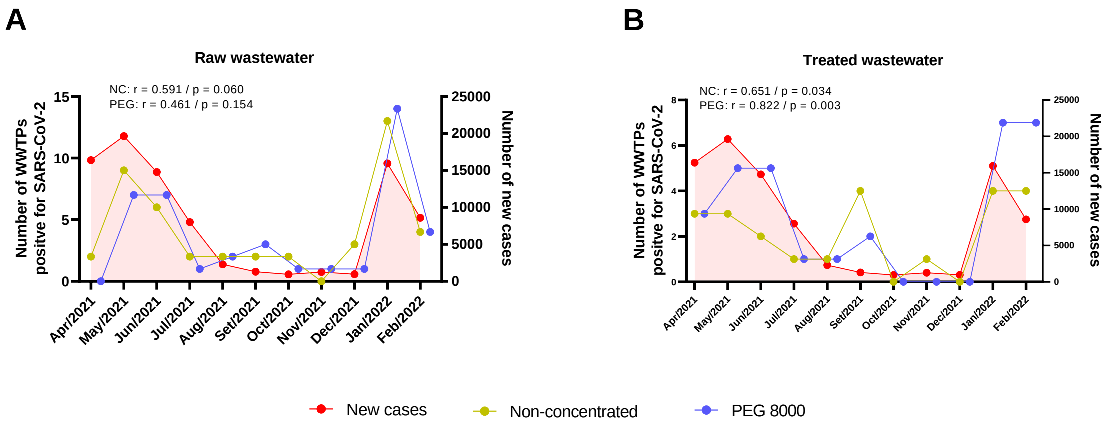

Supplementary Figure S3. Relationship between average new cases of COVID-19 and SARS-CoV-2 detection in samples from different WWTPs of Salvador city. (A) Raw wastewater and (B) treated wastewater samples were collected between Apr/2021 - Jan/2022 and submitted or not to PEG 8000 concentration. SARS-CoV-2 RNA was detected via RT-qPCR using the N2 primer, as described in Methodology section. The correlations between non-concentrated or PEG 8000 concentrated raw wastewater with the number of new cases for COVID-19 and between non-concentrated or PEG 8000 concentrated treated wastewater with the number of new cases for COVID-19 are showed in each graph (A) and (B), respectively. Spearman test was used to verify the correlations. The r and p values are plotted in each graph.

Supplementary Table S1. Results of RT-qPCR assays by month according with viral load.

APRIL 2021					
**Treatment Station**	**Wastewater sample**	**N1 C-**	**eGFP C-**	**N1 C+**	**eGFP C+**
Baixa Fria	Raw	x	9,47E+08	x	8,75E+06
Castelo Branco	Raw	x	7,60E+08	x	1,64E+07
Faz. Grande U11	Raw	x	6,40E+11	x	4,63E+09
Faz. Grande U7	Raw	x	5,69E+11	x	3,30E+09
Irmã Dulce	Raw	x	4,78E+11	**ND**	6,09E+09
Irmã Dulce	Effluent	**ND**	3,60E+11	**7,53E+06**	5,76E+09
Mirante do Trobogy	Raw	**ND**	6,03E+11	x	9,15E+09
Mirante do Trobogy	Effluent	**ND**	4,80E+11	**2,22E+06**	1,62E+10
Mocambo	Raw	**ND**	7,13E+11	x	4,31E+09
Mocambo	Effluent	**ND**	5,80E+11	x	2,51E+10
Paralela Park	Raw	**ND**	2,27E+09	x	x
Paralela Park	Effluent	**ND**	1,73E+11	x	x
Recanto dos Pássaros	Raw	x	3,05E+11	**ND**	5,42E+09
Recanto dos Pássaros	Effluent	**4,41E+06**	2,54E+11	**ND**	1,18E+10
Rio Vermelho	Raw	**ND**	6,13E+11	**3,42E+06**	7,76E+09
Vila do Bosque	Raw	x	1,41E+09	x	9,46E+06
Vila do Bosque	Effluent	**4,76E+06**	5,67E+08	**3,10E+06**	7,14E+06
Vila Rita	Raw	x	2,96E+08	x	x
Vila Rita	Effluent	**8,34E+06**	2,27E+09	**ND**	2,39E+07
Walter Baraúna	Raw	x	2,78E+11	x	x
Walter Baraúna	Effluent	x	2,94E+11	x	x
**MAY 2021**					
**Treatment Station**	**Wastewater sample**	**N1 C-**	**eGFP C-**	**N1 C+**	**eGFP C+**
Baixa Fria	Raw	**9,28E+06**	2,91E+11	x	9,48E+09

Bosque das Mangueiras	Raw	x	4,67E+09	x	8,86E+07
Bosque das Mangueiras	Effluent	x	2,55E+09	x	7,54E+07
Cajazeiras XI	Raw	**1,04E+07**	2,78E+09	**ND**	4,03E+07
Cajazeiras X-II	Raw	**ND**	6,99E+08	x	3,18E+07
Castelo Branco	Raw	**8,58E+06**	3,03E+09	x	x
Faz. Grande U11	Raw	**5,15E+06**	2,03E+11	x	5,18E+09
Faz. Grande U7	Raw	**ND**	3,67E+11	x	5,76E+09
Hildete França	Raw	x		**ND**	2,61E+07
Irmã Dulce	Raw	**ND**	5,02E+11	x	1,12E+10
Irmã Dulce	Effluent	x	2,89E+11	**ND**	8,25E+06
Mirante do Trobogy	Raw	**1,39E+07**	9,78E+11	**2,03E+06**	6,21E+09
Mirante do Trobogy	Effluent	x	5,67E+11	**1,76E+06**	9,89E+09
Mocambo	Raw	x	3,23E+11	x	6,21E+09
Mocambo	Effluent	x	2,45E+11	x	8,34E+09
Nova Esperança	Raw	x	x	x	2,99E+07
Nova Esperança	Effluent	**ND**	3,50E+08	**5,84E+06**	1,02E+07
Paralela Park	Raw	x	2,44E+09	**1,04E+07**	x
Paralela Park	Effluent	**2,42E+07**	3,08E+09	**3,00E+05**	6,44E+09
Recanto dos Pássaros	Raw	**4,93E+09**	4,83E+11	**2,61E+09**	8,34E+09
Recanto dos Pássaros	Effluent	**4,66E+07**	1,04E+12	**2,51E+07**	1,10E+10
Rio Vermelho	Raw	**1,02E+07**	3,17E+11	**2,56E+07**	1,03E+10
Silvio Leal	Raw	**2,66E+07**	5,45E+11	**1,52E+07**	6,68E+09
Silvio Leal	Effluent	**ND**	4,03E+11	**5,80E+06**	1,33E+10
Verde Vida	Raw	x	4,93E+08	**ND**	x
Verde Vida	Effluent	x	4,48E+08	**4,30E+06**	1,10E+07
Vila do Bosque	Raw	x	3,06E+07	x	6,54E+06
Vila do Bosque	Effluent	x	2,94E+11	**5,06E+07**	x
Vivenda de Ipitanga	Raw	x	3,91E+09	x	1,81E+08

Vivenda de Ipitanga	Effluent	x	2,34E+10	x	2,35E+07
Walter Baraúna	Raw	**8,40E+06**	3,29E+09	**4,70E+06**	x
Walter Baraúna	Effluent	**5,80E+07**	1,10E+09	**3,79E+06**	1,48E+07
**JUNE 2021**					
**Treatment Station**	**Wastewater sample**	**N1 C-**	**eGFP C-**	**N1 C+**	**eGFP C+**
Baixa Fria	Raw	**1,01E+06**	1,27E+09	**3,47E+05**	1,11E+07
Bosque das Mangueiras	Raw	x	9,71E+08	x	6,15E+07
Bosque das Mangueiras	Effluent	**ND**	2,62E+09	**2,69E+06**	7,30E+07
Cajazeiras XI	Raw	**ND**	9,73E+08	x	7,51E+06
Cajazeiras X-II	Raw	**1,42E+07**	2,96E+09	**3,09E+06**	4,56E+07
Castelo Branco	Raw	x	4,49E+11	**6,12E+06**	9,52E+09
Eunice Weaver	Raw	x	8,94E+09	x	1,04E+08
Faz. Grande U11	Raw	**ND**	4,39E+11	x	3,47E+09
Faz. Grande U7	Raw	x	2,09E+11	x	6,02E+09
Hildete França	Raw	**8,14E+06**	2,82E+09	x	2,39E+07
Hildete França	Effluent	**ND**	1,85E+09	**ND**	3,34E+07
Irmã Dulce	Raw	x	2,14E+11	x	6,00E+09
Irmã Dulce	Effluent	**ND**	1,82E+11	**ND**	1,77E+07
Mirante do Trobogy	Raw	x	2,53E+11	x	3,42E+09
Mirante do Trobogy	Effluent	**ND**	2,98E+11	**ND**	1,47E+10
Mocambo	Raw	x	1,72E+11	x	1,52E+10
Mocambo	Effluent	x	3,18E+11	x	4,25E+09
Nova Esperança	Raw	x	2,52E+09	x	2,08E+07
Nova Esperança	Effluent	x	9,28E+08	x	5,19E+07
Paralela Park	Raw	**4,04E+06**	1,98E+09	**2,20E+06**	2,51E+07
Paralela Park	Effluent	x	2,20E+09	x	2,72E+07
Recanto dos Pássaros	Raw	x	1,07E+09	x	6,57E+06

Recanto dos Pássaros	Effluent	**2,27E+07**	3,07E+11	**1,23E+07**	1,28E+10
Rio Vermelho	Raw	**1,80E+07**	6,50E+10	**3,04E+07**	1,17E+10
Silvio Leal	Raw	**1,03E+07**	1,70E+11	**4,61E+06**	2,90E+09
Silvio Leal	Effluent	x	1,08E+11	**ND**	3,68E+09
Verde Vida	Raw	**4,21E+06**	x	**2,78E+06**	x
Verde Vida	Effluent	x	x	**4,65E+06**	3,24E+07
Vila do Bosque	Raw	x	1,94E+09	x	3,27E+07
Vila do Bosque	Effluent	x	1,78E+10	**4,19E+05**	6,00E+07
Vila Rita	Raw	x	2,62E+09	**ND**	2,76E+07
Vila Rita	Effluent	x	2,09E+09	x	2,57E+07
Vivenda de Ipitanga	Raw	**ND**	4,65E+09	**1,83E+06**	8,21E+07
Vivenda de Ipitanga	Effluent	**4,76E+06**	4,81E+09	**3,39E+06**	1,33E+10
Walter Baraúna	Raw	**2,93E+07**	1,44E+09	**5,75E+06**	1,86E+07
Walter Baraúna	Effluent	**4,28E+07**	2,04E+09	**3,26E+07**	5,10E+07
**JULY 2021**					
**Treatment Station**	**Wastewater sample**	**N1 C-**	**eGFP C-**	**N1 C+**	**eGFP C+**
Baixa Fria	Raw	x	2,93E+11	x	6,14E+09
Bosque das Mangueiras	Effluent	x	6,85E+09	x	x
Cajazeiras XI	Raw	x	9,43E+08	**ND**	8,76E+06
Cajazeiras X-II	Raw	x	1,50E+09	**ND**	1,14E+07
Castelo Branco	Raw	x	2,49E+11	x	4,69E+09
Eunice Weaver	Raw	x	7,31E+09	x	1,14E+08
Faz. Grande U11	Raw	x	2,20E+11	x	2,00E+09
Faz. Grande U7	Raw	x	2,64E+11	x	3,80E+09
Hildete França	Raw	x	1,89E+09	**2,54E+05**	1,86E+07
Hildete França	Effluent	x	1,08E+09	x	1,47E+07
Irmã Dulce	Raw	x	2,66E+11	**ND**	5,99E+09

Irmã Dulce	Effluent	x	1,69E+11	**3,87E+06**	7,14E+06
Mirante do Trobogy	Raw	**ND**	1,80E+11	x	2,98E+09
Mirante do Trobogy	Effluent	**4,91E+06**	2,40E+11	**1,38E+06**	1,06E+10
Mocambo	Raw	x	2,41E+11	x	3,77E+09
Mocambo	Effluent	x	2,16E+11	x	4,73E+09
Muriçoca	Raw	x	7,83E+09	x	6,53E+07
Muriçoca	Effluent	x	7,44E+09	x	5,33E+07
Nova Esperança	Raw	**ND**	2,49E+09	x	6,20E+07
Nova Esperança	Effluent	**8,67E+07**	1,98E+09	**ND**	5,32E+07
Paralela Park	Raw	x	2,41E+11	x	4,80E+09
Paralela Park	Effluent	x	3,66E+11	x	5,01E+09
Recanto dos Pássaros	Raw	x	8,00E+09	x	6,90E+06
Recanto dos Pássaros	Effluent	x	1,95E+11	**3,80E+06**	7,03E+09
Rio Vermelho	Raw	**ND**	1,37E+11	**2,80E+06**	8,06E+09
Silvio Leal	Raw	x	2,48E+11	x	1,04E+10
Silvio Leal	Effluent	x	1,73E+11	x	1,18E+10
Verde Vida	Raw	**1,64E+07**	2,23E+09	**3,23E+06**	1,29E+07
Verde Vida	Effluent	x	2,93E+11	x	6,14E+09
Vila do Bosque	Raw	x	4,80E+11	x	4,10E+09
Vila do Bosque	Effluent	**ND**	3,10E+11	**ND**	8,37E+09
Vila Rita	Raw	x	5,36E+09	**ND**	1,01E+08
Vila Rita	Effluent	x	2,78E+09	x	3,64E+07
Vivenda de Ipitanga	Raw	x	3,99E+09	x	x
Vivenda de Ipitanga	Effluent	x	3,02E+09	x	3,68E+09
Walter Baraúna	Raw	x	2,40E+11	x	2,33E+09
Walter Baraúna	Effluent	x	2,68E+11	**ND**	4,55E+09
**AUGUST 2021**					

Treatment Station	Wastewater sample	N1 C-	eGFP C-	N1 C+	eGFP C+
Baixa Fria	Raw	x	3,10E+11	x	2,16E+09
Bosque das Mangueiras	Raw	**ND**	2,53E+09	x	5,17E+07
Bosque das Mangueiras	Effluent	**ND**	2,95E+09	x	3,08E+07
Cajazeiras XI	Raw	x	1,27E+09	x	1,27E+08
Cajazeiras X-II	Raw	**8,60E+06**	1,17E+09	x	2,49E+07
Castelo Branco	Raw	x	2,82E+11	x	6,45E+09
Eunice Weaver	Raw	x	2,18E+11	x	1,84E+08
Faz. Grande U11	Raw	x	1,87E+11	**ND**	5,63E+08
Faz. Grande U7	Raw	x	2,71E+11	x	6,11E+09
Hildete França	Raw	x	4,91E+11	**2,40E+06**	4,15E+09
Hildete França	Effluent	**3,33E+06**	7,62E+11	**1,67E+06**	8,59E+09
Irmã Dulce	Raw	x	5,93E+11	x	7,97E+09
Irmã Dulce	Effluent	x	3,36E+11	x	6,77E+09
Mirante do Trobogy	Raw	x	1,85E+11	x	1,96E+09
Mirante do Trobogy	Effluent	**ND**	1,70E+11	**ND**	5,05E+09
Mocambo	Raw	x	3,91E+11	**ND**	8,38E+09
Mocambo	Effluent	x	2,67E+11	x	7,44E+09
Muriçoca	Raw	x	2,79E+11	x	6,88E+09
Muriçoca	Effluent	x	1,77E+11	x	1,70E+10
Nova Esperança	Raw	x	1,69E+09	x	8,00E+07
Nova Esperança	Effluent	x	6,34E+08	**5,23E+06**	6,25E+07
Paralela Park	Raw	x	6,50E+11	**ND**	1,30E+10
Paralela Park	Effluent	**ND**	5,97E+11	x	1,35E+10
Recanto dos Pássaros	Raw	x	6,28E+11	x	1,02E+10
Recanto dos Pássaros	Effluent	x	1,63E+11	**ND**	1,01E+10

Rio Vermelho	Raw	x	3,08E+11	x	1,70E+10
Silvio Leal	Raw	x	1,57E+11	x	9,69E+09
Silvio Leal	Effluent	x	9,65E+10	x	1,10E+10
Verde Vida	Raw	**5,00E+06**	9,86E+10	**ND**	2,39E+08
Verde Vida	Effluent	x	3,64E+11	x	3,18E+09
Vila do Bosque	Raw	x	4,13E+11	x	7,46E+09
Vila do Bosque	Effluent	**ND**	5,46E+11	**ND**	1,81E+10
Vila Rita	Raw	x	3,33E+11	x	3,97E+09
Vila Rita	Effluent	**7,21E+06**	4,91E+11	**2,28E+06**	1,78E+10
Vivenda de Ipitanga	Raw	x	2,99E+09	x	4,29E+07
Vivenda de Ipitanga	Effluent	**ND**	1,84E+09	x	9,86E+09
Walter Baraúna	Raw	x	5,44E+11	x	6,01E+09
Walter Baraúna	Effluent	x	4,79E+11	x	3,40E+09
**SEPTEMBER 2021**					
**Treatment Station**	**Wastewater sample**	**N1 C-**	**eGFP C-**	**N1 C+**	**eGFP C+**
Baixa Fria	Raw	**ND**	3,85E+11	x	1,65E+10
Bosque das Mangueiras	Raw	x	7,45E+11	x	1,00E+10
Bosque das Mangueiras	Effluent	x	3,82E+11	x	1,09E+10
Cajazeiras XI	Raw	**7,85E+07**	3,97E+11	**4,43E+07**	8,90E+09
Cajazeiras X-II	Raw	x	4,07E+11	x	7,96E+09
Castelo Branco	Raw	x	3,51E+11	x	8,95E+09
Eunice Weaver	Raw	x	3,13E+11	x	3,91E+09
Faz. Grande U11	Raw	x	3,11E+11	x	2,24E+09
Faz. Grande U7	Raw	x	2,88E+11	x	5,16E+09
Hildete França	Raw	x	4,19E+11	x	2,14E+09
Hildete França	Effluent	x	3,83E+11	x	2,07E+10
Irmã Dulce	Raw	x	3,19E+11	x	9,35E+09

Irmã Dulce	Effluent	**1,26E+07**	4,30E+11	**ND**	6,00E+07
Mirante do Trobogy	Raw	x	3,09E+11	x	3,93E+09
Mirante do Trobogy	Effluent	**ND**	1,76E+12	**2,40E+06**	2,07E+10
Mocambo	Raw	x	3,60E+11	**ND**	8,87E+09
Mocambo	Effluent	x	2,26E+11	x	9,03E+09
Muriçoca	Raw	**9,33E+06**	4,85E+11	x	2,03E+09
Muriçoca	Effluent	x	3,66E+11	**ND**	9,32E+09
Nova Esperança	Raw	x	7,85E+11	x	1,44E+10
Nova Esperança	Effluent	x	6,40E+11	x	3,83E+09
Paralela Park	Raw	x	2,44E+11	x	9,37E+09
Paralela Park	Effluent	x	3,12E+11	x	1,70E+10
Recanto dos Pássaros	Raw	x	2,47E+11	x	7,41E+09
Recanto dos Pássaros	Effluent	**1,06E+07**	2,07E+11	**7,22E+06**	8,15E+09
Rio Vermelho	Raw	x	1,85E+11	**3,32E+06**	2,81E+10
Silvio Leal	Raw	x	3,92E+11	x	1,27E+10
Silvio Leal	Effluent	x	2,95E+11	x	1,28E+10
Verde Vida	Raw	**5,58E+06**	2,25E+11	**ND**	5,72E+09
Verde Vida	Effluent	x	5,31E+11	x	1,93E+10
Vila do Bosque	Raw	x	4,38E+11	x	7,59E+09
Vila do Bosque	Effluent	x	2,12E+11	**3,06E+06**	1,15E+10
Vila Rita	Raw	x	2,61E+11	**ND**	2,43E+09
Vila Rita	Effluent	**6,07E+06**	2,42E+11	**2,54E+06**	1,22E+10
Vivenda de Ipitanga	Raw	x	4,09E+11	x	3,90E+09
Vivenda de Ipitanga	Effluent	x	5,69E+11	x	3,51E+08
Walter Baraúna	Raw	x	9,83E+11	x	3,63E+09
Walter Baraúna	Effluent	x	5,33E+11	x	5,13E+09
**OCTOBER 2021**					

Treatment Station	Wastewater sample	N1 C-	eGFP C-	N1 C+	eGFP C+
Baixa Fria	Raw	**ND**	7,00E+11	x	2,77E+09
Bosque das Mangueiras	Raw	x	1,41E+09	x	5,15E+08
Bosque das Mangueiras	Effluent	x	1,27E+09	x	2,83E+08
Cajazeiras XI	Raw	x	9,97E+08	x	x
Cajazeiras X-II	Raw	**ND**	8,37E+08	x	8,19E+06
Castelo Branco	Raw	x	1,54E+11	x	9,53E+09
Eunice Weaver	Raw	x	3,00E+09	x	x
Faz. Grande U11	Raw	x	4,57E+11	x	2,60E+09
Faz. Grande U7	Raw	**ND**	3,71E+11	**ND**	8,04E+09
Hildete França	Raw	x	3,60E+09	x	1,32E+08
Hildete França	Effluent	x	1,22E+09	x	6,39E+07
Irmã Dulce	Raw	x	5,16E+11	x	7,25E+09
Irmã Dulce	Effluent	**ND**	3,19E+11	**ND**	8,37E+09
Mirante do Trobogy	Raw	**2,49E+07**	2,47E+11	x	1,33E+10
Mirante do Trobogy	Effluent	x	7,00E+11	**ND**	1,34E+10
Mocambo	Raw	x	2,32E+11	x	9,58E+09
Mocambo	Effluent	x	4,42E+11	x	1,37E+10
Muriçoca	Raw	x	8,09E+09	x	6,47E+07
Muriçoca	Effluent	x	7,69E+09	x	2,18E+08
Nova Esperança	Raw	x	1,37E+09	x	8,01E+07
Nova Esperança	Effluent	x	6,21E+08	**ND**	2,33E+07
Paralela Park	Raw	x	8,50E+11	x	6,70E+09
Paralela Park	Effluent	x	9,56E+11	x	9,30E+09
Recanto dos Pássaros	Raw	x	8,15E+11	x	4,32E+09
Recanto dos Pássaros	Effluent	**ND**	1,18E+11	x	x
Rio Vermelho	Raw	x	2,49E+11	**ND**	6,99E+09
Silvio Leal	Raw	x	5,69E+11	x	5,45E+09

Silvio Leal	Effluent	x	2,28E+11	x	7,03E+09
Verde Vida	Raw	**2,25E+07**	4,70E+08	**4,62E+06**	x
Verde Vida	Effluent	x	2,69E+09	**ND**	7,98E+07
Vila do Bosque	Raw	x	1,53E+11	x	x
Vila do Bosque	Effluent	x	2,63E+12	**ND**	9,98E+09
Vila Rita	Raw	x	1,51E+09	x	4,15E+07
Vila Rita	Effluent	x	1,96E+09	**7,91E+05**	5,02E+07
Vivenda de Ipitanga	Raw	x	3,71E+09	x	3,02E+07
Vivenda de Ipitanga	Effluent	x	3,88E+10	x	1,20E+10
Walter Baraúna	Raw	x	4,03E+11	x	9,14E+09
Walter Baraúna	Effluent	x	7,65E+11	x	8,36E+09
**NOVEMBER 2021**					
**Treatment Station**	**Wastewater sample**	**N1 C-**	**eGFP C-**	**N1 C+**	**eGFP C+**
Bosque das Mangueiras	Raw	x	2,77E+09	x	x
Bosque das Mangueiras	Effluent	x	4,47E+10	x	4,07E+07
Cajazeiras XI	Raw	x	1,07E+09	x	8,01E+07
Castelo Branco	Raw	x	2,05E+11	x	1,23E+10
Faz. Grande U11	Raw	x	2,76E+09	x	2,67E+09
Faz. Grande U7	Raw	x	3,79E+09	x	4,12E+09
Hildete França	Raw	x	1,86E+09	**ND**	x
Hildete França	Effluent	x	2,14E+09	x	9,25E+07
Irmã Dulce	Raw	x	1,60E+11	**ND**	1,08E+10
Irmã Dulce	Effluent	**ND**	2,67E+11	**1,05E+07**	1,81E+10
Mirante do Trobogy	Raw	x	2,81E+09	x	1,98E+07
Mirante do Trobogy	Effluent	x	2,66E+08	x	9,32E+06
Mocambo	Raw	x	2,22E+09	x	2,95E+09
Mocambo	Effluent	**ND**	3,06E+09	x	4,46E+09
Nova Esperança	Raw	**9,53E+06**	1,05E+09	x	3,17E+07

Nova Esperança	Effluent	x	3,06E+08	**ND**	1,35E+07
Paralela Park	Raw	x	3,85E+11	x	x
Paralela Park	Effluent	x	3,44E+11	x	x
Recanto dos Pássaros	Raw	**ND**	3,05E+09		8,05E+07
Recanto dos Pássaros	Effluent	**ND**	2,83E+09	**1,86E+06**	4,44E+09
Rio Vermelho	Raw	**3,19E+06**	3,11E+09	x	1,97E+07
Silvio Leal	Raw	x	2,85E+11	x	7,93E+09
Silvio Leal	Effluent	x	1,58E+11	x	1,01E+10
Verde Vida	Raw	**1,16E+07**	3,62E+08	x	x
Verde Vida	Effluent	**ND**	5,15E+09	**ND**	6,49E+07
Vila do Bosque	Raw	x	2,65E+11	x	x
Vila do Bosque	Effluent	x	2,08E+11	x	x
Vila Rita	Raw	**1,78E+08**	3,23E+09	x	x
Vila Rita	Effluent	x	3,37E+09	**ND**	8,43E+07
Vivenda de Ipitanga	Raw	x	1,29E+09	x	5,41E+07
Vivenda de Ipitanga	Effluent	x	1,22E+09	x	4,78E+09
Walter Baraúna	Raw	x	1,01E+09	x	x
Walter Baraúna	Effluent	x	1,14E+09	x	3,08E+07
**DECEMBER 2021**					
**Treatment Station**	**Wastewater sample**	**N1 C-**	**eGFP C-**	**N1 C+**	**eGFP C+**
Bosque das Mangueiras	Raw	x	1,29E+09	x	2,88E+08
Bosque das Mangueiras	Effluent	**5,04E+05**	1,84E+09	x	1,39E+08
Cajazeiras XI	Raw	x	7,03E+09	x	9,96E+07
Cajazeiras X-II	Raw	**1,41E+07**	1,66E+09	x	3,79E+07
Castelo Branco	Raw	**3,23E+06**	5,91E+08	x	4,05E+09
Faz. Grande U11	Raw	x	1,08E+09	x	2,27E+07
Faz. Grande U7	Raw	x	1,07E+09	x	1,63E+07
Hildete França	Raw	x	2,23E+09	x	2,95E+07
Hildete França	Effluent	x	8,73E+08	x	2,35E+07

Irmã Dulce	Raw	**ND**	x	x	4,23E+09
Irmã Dulce	Effluent	**8,16E+06**	9,26E+08	x	1,15E+10
Mirante do Trobogy	Raw	x	6,93E+08	x	1,96E+07
Mirante do Trobogy	Effluent	x	1,21E+09	x	2,17E+07
Mocambo	Raw	x	1,24E+09	x	1,83E+07
Mocambo	Effluent	x	9,61E+08	x	2,77E+07
Nova Esperança	Raw	x	2,55E+09	x	5,06E+07
Nova Esperança	Effluent	x	1,35E+09	**1,29E+06**	2,63E+07
Paralela Park	Raw	x	x	x	4,83E+09
Paralela Park	Effluent	x	3,66E+09	x	5,86E+09
Recanto dos Pássaros	Raw	**ND**	2,13E+11	x	3,08E+09
Recanto dos Pássaros	Effluent	x	1,44E+11	x	5,76E+09
Rio Vermelho	Raw	**ND**	8,60E+08	**1,76E+06**	2,07E+07
Silvio Leal	Raw	**8,22E+06**	2,93E+09	x	1,00E+07
Silvio Leal	Effluent	x	3,16E+07	x	8,15E+09
Verde Vida	Raw	x	2,58E+09	x	4,38E+07
Verde Vida	Effluent	x	1,84E+09	**ND**	7,16E+07
Vila do Bosque	Raw	**1,65E+07**	4,57E+10	x	x
Vila do Bosque	Effluent	x	6,13E+10	x	x
Vila Rita	Raw	x	2,52E+09	x	2,87E+07
Vila Rita	Effluent	**5,30E+06**	2,42E+09	x	1,91E+07
Vivenda de Ipitanga	Raw	x	1,22E+09	x	1,42E+08
Vivenda de Ipitanga	Effluent	x	9,68E+08	x	8,13E+08
Walter Baraúna	Raw	x	2,90E+08	x	x
Walter Baraúna	Effluent	**ND**	7,63E+08	**ND**	1,28E+07
**JANUARY 2022**					
**Treatment Station**	**Wastewater sample**	**N1 C-**	**eGFP C-**	**N1 C+**	**eGFP C+**

Bosque das Mangueiras	Raw	**ND**	6,13E+08	x	2,01E+07
Bosque das Mangueiras	Effluent	**ND**	1,08E+09	x	8,59E+07
Cajazeiras XI	Raw	**2,17E+08**	5,69E+08	**9,94E+07**	x
Cajazeiras X-II	Raw	**2,71E+07**	1,01E+09	x	4,01E+07
Castelo Branco	Raw	**ND**	2,79E+11	x	2,34E+07
Faz. Grande U11	Raw	**1,28E+07**	8,37E+08	x	5,81E+07
Faz. Grande U7	Raw	**1,47E+08**	1,12E+09	x	6,27E+07
Hildete França	Raw	x	2,38E+09	x	2,95E+07
Hildete França	Effluent	x	5,02E+08	x	x
Irmã Dulce	Raw	**ND**	4,28E+11	**ND**	x
Irmã Dulce	Effluent	x	1,73E+09	x	9,98E+09
Mirante do Trobogy	Raw	**1,72E+07**	3,05E+11	x	4,36E+07
Mirante do Trobogy	Effluent	**3,37E+07**	6,61E+12	x	x
Mocambo	Raw	**6,90E+07**	3,01E+08	x	1,05E+08
Mocambo	Effluent	x	4,28E+09	x	x
Muriçoca	Raw	**4,63E+06**	3,40E+09	**1,08E+06**	9,87E+07
Nova Esperança	Raw	x	1,78E+11	x	x
Nova Esperança	Effluent	x	2,66E+08	x	1,68E+08
Paralela Park	Raw	**1,85E+07**	3,85E+08	x	4,06E+07
Paralela Park	Effluent	x	7,03E+08	**ND**	1,26E+08
Recanto dos Pássaros	Raw	x	1,11E+09	x	2,19E+07
Recanto dos Pássaros	Effluent	**1,02E+07**	5,41E+08	x	x
Rio Vermelho	Raw	**1,21E+09**	1,84E+11	x	3,26E+07
Silvio Leal	Raw	x	1,92E+09	**ND**	x
Silvio Leal	Effluent	x	1,32E+09	x	x
Verde Vida	Raw	x	2,24E+10	x	2,37E+07
Verde Vida	Effluent	**1,27E+07**	9,10E+08	**6,43E+06**	5,07E+07
Vila do Bosque	Raw	x	1,40E+09	**4,10E+06**	1,83E+08
Vila do Bosque	Effluent	**1,63E+06**	9,53E+08	**4,94E+06**	1,15E+09

Vila Rita	Raw	**ND**	2,31E+09	**ND**	6,97E+07
Vila Rita	Effluent	**1,38E+07**	1,94E+09	**7,50E+05**	4,27E+07
Vivenda de Ipitanga	Raw	**4,85E+07**	1,18E+09	**4,05E+07**	1,34E+08
Vivenda de Ipitanga	Effluent	**5,01E+07**	1,14E+09	**6,67E+07**	2,81E+08
Walter Baraúna	Raw	x	2,39E+09	x	x
Walter Baraúna	Effluent	**ND**	1,33E+09	**1,86E+07**	x
**FEBRUARY 2022**					
**Treatment Station**	**Wastewater sample**	**N1 C-**	**eGFP C-**	**N1 C+**	**eGFP C+**
Bosque das Mangueiras	Raw	x	3,11E+09	x	3,49E+07
Bosque das Mangueiras	Effluent	**ND**	4,81E+09	x	1,28E+08
Cajazeiras XI	Raw	**2,79E+06**	7,32E+08	**ND**	9,58E+07
Castelo Branco	Raw	**ND**	1,97E+09	x	5,17E+07
Faz. Grande U11	Raw	x	4,92E+09	x	4,00E+07
Faz. Grande U7	Raw	x	3,86E+09	x	2,98E+07
Hildete França	Raw	x	8,52E+08	x	3,72E+07
Hildete França	Effluent	**1,08E+07**	2,26E+09	**6,11E+05**	x
Irmã Dulce	Raw	**ND**	4,64E+10	**1,34E+06**	7,35E+07
Irmã Dulce	Effluent	**5,53E+07**	4,70E+09	**1,94E+06**	4,60E+07
Mirante do Trobogy	Raw	x	2,43E+09	x	x
Mirante do Trobogy	Effluent	x	4,34E+09	x	1,09E+08
Mocambo	Raw	x	4,58E+09	x	5,21E+07
Mocambo	Effluent	x	4,45E+09	x	6,28E+07
Nova Esperança	Raw	x	x	x	1,66E+07
Nova Esperança	Effluent	x	2,68E+08	x	1,15E+07
Paralela Park	Raw	**ND**	6,76E+08	**ND**	x
Paralela Park	Effluent	**1,72E+07**	2,25E+10	x	2,50E+07
Recanto dos Pássaros	Raw	**ND**	1,17E+09	x	x
Recanto dos Pássaros	Effluent	**2,27E+07**	1,94E+09	**8,22E+06**	1,77E+07
Rio Vermelho	Raw	x	3,72E+09	x	4,34E+07

Silvio Leal	Raw	**1,82E+07**	2,51E+09	**4,38E+06**	2,32E+07
Silvio Leal	Effluent	**6,00E+07**	2,77E+09	x	4,44E+09
Verde Vida	Raw	x	x	x	x
Verde Vida	Effluent	x	6,32E+08	**6,02E+06**	1,04E+07
Vila do Bosque	Raw	x	5,07E+08	x	x
Vila do Bosque	Effluent	**1,95E+07**	1,02E+09	**1,53E+07**	2,35E+07
Vivenda de Ipitanga	Raw	**ND**	2,23E+09	x	5,81E+07
Vivenda de Ipitanga	Effluent	x	2,84E+09	x	1,15E+09
Walter Baraúna	Raw	**8,72E+06**	6,52E+08	x	x
Walter Baraúna	Effluent	**ND**	5,17E+08	**6,22E+06**	2,68E+07
